# Random clinical trial to evaluate the effect of a multimodal intervention in hand hygiene in primary care in Madrid

**DOI:** 10.1186/1753-6561-5-S6-P257

**Published:** 2011-06-29

**Authors:** A Cañada, Carmen Martín, Sonia Soto, Juan Carlos Abánades, Miguel Salinero

**Affiliations:** 1 Gerencia de Atención Primaria, Servico Madrileño de la Salud, Madrid, Spain

## Introduction / objectives

Objective was to evaluate the effectiveness of multimodal intervention, in primary health care professionals, to improve compliance with practice of hand hygiene, based on WHO's 5 moments.

## Methods

Double blind, parallel group clinical trial with control group (CG), randomized by cluster. Performed at 21 primary health care centers, during 2009-2010, with 214 health professionals.

Hand hygiene compliance level was evaluated at the moment basal and six months after the intervention, by a single external observer. Professionals ignored in which activity they were being observed, previously signed an informed consent.

Variables:related to WHO's 5 moments and with professionals (profession, sex, type of contract and years of experience).

Multimodal intervention carried out with intervention group (IG), consisted of a theoretical-practical workshop in four sessions, providing the visiting room with hydro-alcoholic solutions and reminder signs.

Statistical analysis: descriptive analysis, Student’s t for independent samples, and the Mann-Whitney U or the Kruskal-Wallis test and multiple linear regression techniques have been utilized to analyze baseline compliance.

## Results

Study was completed by 170 professionals: 84 (IG), 86 (CG), with no differences in the baseline characteristics. Professionals in the intervention group increased their level of compliance for hand hygiene by 21.6 points (CI95% from 13.83 to 28.48), compared with the control group. Moment 1 showed the highest increase in improvement (9.5 points). The level of compliance basal was 8.1% (CI95% from 6.2 to 10.1). More than 20 years of job experience is significantly associated with very low levels of compliance.

## Conclusion

Compliance with hand hygiene can be improved with a multimodal intervention, fundamentally training. It provides a valid methodology to other health centers.

## Disclosure of interest

None declared.

